# Reply to Corrales-García, E.M.; Delgado-López, P.D. Comment on “Giotta Lucifero et al. Impact of Irradiation on Post-Surgical Residuals of WHO Grade I Meningioma. *J. Clin. Med.* 2025, *14*, 5829”

**DOI:** 10.3390/jcm14248800

**Published:** 2025-12-12

**Authors:** Alice Giotta Lucifero, Rami Almefty, Ossama Al-Mefty

**Affiliations:** 1 Department of Neurosurgery, Mass General Brigham, Harvard Medical School, Boston, MA 02115, USA; 2 Department of Brain and Behavioral Sciences, University of Pavia, 27100 Pavia, Italy; 3 Department of Neurosurgery, Lewis Katz School of Medicine, Temple University, Philadelphia, PA 19140, USA

We greatly appreciate the insightful comments from Dr Corrales García and Dr. Delgado López [1]. We are in full agreement and acknowledge the shortcomings in the manuscript that they have raised concerning this retrospective observational study [2].

The size, location, and recurrent nature of the tumors are presented in the data and accounted for. As a matter of fact, the size is one of the persistent adverse indicators of outcome.

We presented the radiotherapy data by modality and protocol as they are in the record. We analyzed each modality individually (stereotactic fractionated radiation therapy, radiosurgery, and proton beam) and found that all were risk factors for recurrence, and that stereotactic fractionated radiation therapy and radiosurgery were also risk factors for cause-specific death.

The concept of “clinical control” versus “radiological control” is well established in tumor oncology [3]. It is applied in outcome studies of radiated meningiomas [4,5] and specifically in gamma knife radiosurgery outcomes, as Kondziolka et al. stated “clinical tumor control rate (no resection required)” [6]. It is mainly used to indicate that the tumor is still under control despite its growth. Thus, we analyzed and reported both, as is common in similar articles.

We concur with Dr. Corrales García and Dr. Delgado López on the well-known effect of radiation on DNA and tumor progression, which our study shows to be very significant in meningiomas.

We are very cognizant of and cited some of the conspicuous literature that shows superlative outcomes of the modern radiation therapy on WHO grade I meningioma, including three of the references kindly provided by Dr. Corrales García and Dr. Delgado López.

We have the same reservation about the article by Alfredo et al., with a short follow-up of a median of 36 months [7].

Unfortunately, there has not been a prospective double-blinded “level I evidence-based” study on the effect of radiation on WHO grade I. The prospective study cited only addresses the outcome of WHO grade I meningiomas that had not received irradiation [8].

As we indicated, the length of follow-up is the critical factor that explains the contradictions between the results of the relatively short follow-up in comparison to our extended long-term follow-up. We believe that for WHO grade I meningioma studies, a minimum of 10 years needs to pass.
